# Analysis of cell wall synthesis and metabolism during early germination of *Blumeria graminis* f. sp. *hordei* conidial cells induced *in vitro*

**DOI:** 10.1016/j.tcsw.2019.100030

**Published:** 2019-08-14

**Authors:** Trang A.T. Pham, Julian G. Schwerdt, Neil J. Shirley, Xiaohui Xing, Yves S.Y. Hsieh, Vaibhav Srivastava, Vincent Bulone, Alan Little

**Affiliations:** a ARC Centre of Excellence in Plant Cell Walls, School of Agriculture, Food and Wine, University of Adelaide, Waite Campus, Glen Osmond, SA 5064, Australia; b Adelaide Glycomics, School of Agriculture, Food and Wine, University of Adelaide, Waite Campus, Glen Osmond, SA 5064, Australia; c Division of Glycoscience, School of Engineering Sciences in Chemistry, Biotechnology and Health, Royal Institute of Technology (KTH), AlbaNova University Centre, 106 91 Stockholm, Sweden

**Keywords:** *Blumeria graminis* f. sp. *hordei*, Cell wall, Metabolism, Pathogenesis, Pre-penetration

## Abstract

As an obligate biotroph, *Blumeria graminis* f. sp. *hordei* (*Bgh*) cannot be grown in an axenic culture, and instead must be cultivated on its host species, *Hordeum vulgare* (barley). In this study an *in vitro* system utilizing *n*-hexacosanal, a constituent of the barley cuticle and known inducer of *Bgh* germination, was used to cultivate *Bgh* and differentiate conidia up to the appressorial germ tube stage for analysis. Transcriptomic and proteomic profiling of the appressorial germ tube stage revealed that there was a significant shift towards energy and protein production during the pre-penetrative phase of development, with an up-regulation of enzymes associated with cellular respiration and protein synthesis, modification and transport. Glycosidic linkage analysis of the cell wall polysaccharides demonstrated that during appressorial development an increase in 1,3- and 1,4-linked glucosyl residues and xylosyl residues was detected along with a significant decrease in galactosyl residues. The use of this *in vitro* cultivation method demonstrates that it is possible to analyse the pre-penetrative processes of *Bgh* development in the absence of a plant host.

## Introduction

The barley powdery mildew, *Blumeria graminis* f. sp. *hordei* (*Bgh*), is a widespread pathogen that causes important damage to barley crops. Like all powdery mildews *Bgh* is an obligate biotrophic pathogen, meaning it cannot be grown *in vitro* and is entirely dependent on the living host for growth and propagation.

Failure to control outbreaks can result in widespread damage and severe harvest losses. Disease control is implemented by the routine use of fungicides and intensive breeding of novel resistant varieties. However, the resilience of *Bgh* has allowed the fungus to rapidly develop fungicide resistance and overcome breed resistance (Wolfe, 1984, Wolfe and Mcdermott, 1994, Chin et al., 2001). New approaches and techniques are required if *Bgh* is to be effectively controlled. This involves the development of new classes of fungicides and the introgression of novel disease resistance genes. To develop these approaches a finer understanding of the molecular and physiological changes that occur in *Bgh* during conidial germination and pathogenesis is required.

The asexual lifestyle of *Bgh* proceeds in a highly controlled and synchronous manner (Both et al., 2005). Pathogenesis starts when an airborne conidium makes contact with the leaf surface. The conidium subsequently produces an extracellular matrix and a short primary germ tube emerges (Edwards, 2002). Shortly after, a secondary germ tube emerges which develops into an appressorial germ tube (agt). A penetration peg forms under the appressorium and forces its way through the host cell wall with a combination of enzymatic degradation and turgor pressure generated in the appressorium (Pryce-Jones et al., 1999). A haustorium develops in the periplasmic space of the epidermal cell and allows the *Bgh* cell to take up nutrients from the host. Once a functional haustorium is established epiphytic mycelium proliferates and assembles additional secondary appressoria that breach the cells and form more haustoria.

The *Bgh* pathogen is one of the most intensively studied fungal powdery mildews. In spite of its economic and agricultural significance, analysis at the molecular level has lagged considerably compared to other filamentous pathogenic fungi (Zhang et al., 2005). As an obligate biotroph *Bgh* cannot be cultured *in vitro*, severely limiting the repertoire of classical molecular techniques that can be employed, such as genetic manipulation. Standard extraction of *Bgh* material relies on stripping the fungal cells from plant tissues coated with nail polish for examination (Both et al., 2005, Zhang et al., 2000, Zeng et al., 2017). Although this technique is *in planta* it reduces the sensitivity and reliability of many assays due to contamination from the host plant. In addition, the nail polish contains cellulose acetate, which interferes significantly with downstream cell wall analyses.

Only a few studies have focused on the pre-penetrative events that occur before the *Bgh* pathogen infects the host, with most other research focusing on resistance responses of the plant host. Two studies have examined the global gene expression throughout the *Bgh* asexual development cycle, from the ungerminated conidia all the way to maturity at five days post infection, i.e. when conidiophores start to develop (Both et al., 2005, Both et al., 2005). One study focused on the dynamic changes that occur during primary metabolism and found that several glycolytic enzymes were co-ordinately up-regulated during the development of the appressoria while transcripts for lipid degradation were abundant in the conidia and diminished significantly during the later developmental stages (Both et al., 2005). A second study focused on potential virulent determinants and revealed that transcripts related to protein biosynthesis exhibited a substantial increase in expression during post-penetrative stages (Both et al., 2005).

Analyses of the proteomic profile of *Bgh* have revealed that the vast majority of the conidial proteins are classified as metabolic proteins, involved in carbohydrate, lipid, amino acid, and protein metabolism (Noir et al., 2009, Bindschedler et al., 2009). Similar profiles have been observed in other phytopathogenic spores and it has been suggested that these structures are mobile protein factories that are primed to start germination immediately upon physical contact without needing to expend the limited cellular energy resources on *de novo* synthesis (Cooper et al., 2007).

The pre-penetrative processes during the early stages of infection are essential periods of the *Bgh* lifecycle. The early stages of pathogenesis require recognising the plant surface, growing to a suitable penetration point, developing the right infection structures to overcome the host barriers made up of the cuticular layer and the plant cell wall, followed by successfully establishing a haustorium feeding structure. Characterising the complex physiological and molecular changes that occur during the pre-penetrative phases before the *Bgh* has invaded the host is an important step towards disease control.

Our separate analysis of the *Bgh* conidial cell wall revealed that the cell wall is predominantly composed of polysaccharides containing glucosyl residues (63.1%) and a greater proportion of galactopyranosyl residues compared to other species. In addition, trace amounts of xylosyl residues were detected in the cell wall, which is an unusual observation in ascomycetes (accompanying paper in this issue of the journal (Pham et al., 2019)). Annotation of the Carbohydrate Active enZymes (CAZy) in the *Bgh* genome revealed several more CAZy genes than was previously identified (Spanu et al., 2010, de Witt et al., 2012, Hacquard et al., 2013) and has also identified several key enzymes involved in cell wall metabolism. The cell wall is a dynamic structure, continuously expanding and changing throughout the fungal life cycle. To understand these dynamic changes the processes involved in cell wall metabolism need to be examined during key stages of pathogenesis.

The cuticular waxes that cover the surfaces of aerial plant tissues are well known to contain substances that affect the germination and differentiation of several pathogenic plant fungi, including *Bgh* (Carver and Thomas, 1990, Tsuba et al., 2002, Gniwotta et al., 2005, Mendoza-Mendoza et al., 2009). *n*-Hexacosanal, an aldehyde and minor constituent of the barley leaf cuticle, is a strong inducer of germination and differentiation of *Bgh* cells (Tsuba et al., 2002, Hansjakob et al., 2010). An *in vitro* system to cultivate the pre-penetrative stages of *Bgh* using *n*-hexacosanal was developed by Hansjakob et al. (2010) and used to investigate if appressorium morphogenesis and cell cycle progression were linked.

In this study we have adapted the method of Hansjakob et al. (2010) to analyse the molecular events that occur during *Bgh* pre-penetrative development *in vitro*. *Bgh* conidia at specific developmental stages were able to be collected without any contamination from the host plant for analysis of the fungal cell wall composition and associated transcriptomic and proteomic changes.

## Materials and methods

### *In vitro* growth of *Bgh*

The *Bgh* isolate was donated by Professor Richard Oliver (Curtin University, W.A., Australia) and maintained as described in the accompanying paper of this issue of the journal (Pham et al., 2019). Conidial cells of *Bgh* (Pham et al., 2019) were cultivated *in vitro* on 2% agar coated with a Formvar® resin [0.5%]/*n*-hexacosanal [7 × $10^{-4}$ mol/L] membrane and harvested after 2 h, 4 h, and 6 h of incubation. This method was adapted from Hansjakob et al. (2010). Synthesis of *n*-hexacosanal was conducted as previously described by Hansjakob et al. (2010).

### Preparation of *Bgh* cell wall samples and glycosidic linkage analysis

The fungal samples were snap frozen in liquid nitrogen and freeze dried. Glycosidic linkage analyses were performed on conidia and germinating conidial cells as described in Pham et al. (2019) and the data are reported as the mean ±SE. P values were calculated by paired sample t-test and considered significant when <0.05.

### Gene expression analysis by RNA-Seq and qPCR

Frozen ungerminated *Bgh* conidia (0 h) and germinated *Bgh* cells (2 h, 4 h and 6 h) on *n*-hexacosanal/Formvar® resin were ground in liquid nitrogen with a mortar and pestle. Total RNA was extracted using the Plant Total RNA Kit (Sigma-Aldrich) following the manufacturer’s instructions. RNA-Seq analysis and library preparation were performed at the Australian Genome Research Facility (AGRF Ltd., Melbourne, Australia) and sequence reads were quality trimmed and mapped to the *Bgh* DH14 BluGen ( www.blugen.org/) genome assembly version 3.0 as described in Pham et al. (2019). Transcriptome analysis was conducted as in Pham et al. (2019). Mean gene expression from each sample was expressed as reads per kilobase of transcript per million mapped reads (RPKM). Sequence read data will become available at the NCBI sequence read archive (PRJNA557765) upon publication.

The relative expression levels of 13 cell wall related genes were verified by qPCR analysis as described in Pham et al. (2019) using the gene-specific primers listed in Supplementary Table S1. The data were normalised against the geometric mean of four housekeeping genes, namely actin (*bgh00992*), glyceraldehyde 3-phosphate dehydrogenase (*bgh00075*), tubulin (*bgh01972*) and ubiquitin (*bgh03777*).

### Protein extraction and sample preparation for proteomics

The protein extraction method used was adapted from Noir et al. (2009). Approximately 200 mg of 0 h conidia and 6 h germinated *Bgh* were ground in liquid nitrogen with a mortar and pestle. Samples were then suspended in 1 ml of extraction buffer [50 mM Tris-Base pH 8.0, 10 mM ethylenediaminetetraacetic acid (EDTA), 0.5% 3-[(3-cholamidopropyl) dimethylammonio]-1-propanesulphonate (CHAPS), 10 mM dithiothreitol (DTT), 1x cOmplete^TM^EDTA-free Protease Inhibitor Cocktail (Sigma-Aldrich)]. Protein extraction was carried out by five repetitions of vortexing for 30 s, with short pauses on ice for 30 s in between. The mixture was centrifuged at 16,000 g for 5 min at 4 °C, and the supernatant was removed and stored on ice. The extraction procedure was repeated on the pellet using 500 μl of extraction buffer. The supernatant was then freeze-dried and stored at −20 °C.

Proteins were resuspended in denaturing buffer [3% sodium deoxycholate (SDC), 100 mM tetraethylammonium bromide (TEAB)], loaded onto Microcon YM-10 filters and centrifuged for 15 min at 12,000 rpm at room temperature. All subsequent centrifugations involving the YM-10 filters were performed at 12,000 rpm at room temperature. Two hundred and fifty μl of reducing solution [20 mM DTT, 3% SDC, 100 mM TEAB] was added to the filters and the mixtures were incubated at 60 °C for 1 h. One hundred μl of alkylation solution [80 mM iodoacetamide, 3% SDC, 100 mM TEAB] was added and alkylation of the reduced proteins proceeded at 37 °C in the dark for 30 min. The mixture was then centrifuged for 20 min and 300 μl of 100 mM TEAB was added to the filters. The samples were centrifuged for 15 min and the procedure was repeated. Three hundred μl of 100 mM TEAB containing trypsin at an enzyme-to-substrate ratio of 1:50 was added to the filters and the preparations were incubated overnight at 37 °C. The solutions were then centrifuged for 10 min to extract the peptide generated by trypsin. Fifty μl of 100 mM TEAB was added to the filters and spun for 10 min. The procedure was repeated 3 more times to extract all of the peptides.

The total pooled peptide fractions were acidified by adding TFA to a final concentration of 0.5%. The solutions were then vortexed, causing the SDC to precipitate, and the samples were centrifuged at 14,000 rpm for 10 min to remove the SDC pellet. The supernatant was transferred to a new column and peptides were purified using a pep clean column. The eluate was dried under vacuum (SpeedVac) and the peptides were resuspended in 10 μl of 0.1% formic acid and stored at −20 °C.

### Nano-LC-MS/MS analysis of samples subjected to iTRAQ labelling

The peptide samples were analysed after iTRAQ labelling as previously described (Srivastava et al., 2013, Srivastava et al., 2018). Peptide analysis was performed by reverse-phase liquid chromatography-electrospray ionization-tandem mass spectrometry (LC-ESI-MS/MS) using a nanoACQUITY Ultra Performance Liquid Chromatography system coupled to a Q-TOF mass spectrometer. iTraq labelling was performed as described in Srivastava et al. (2013). The peptide fractions were resuspended in 0.1% TFA, loaded onto a C18 trap column (Symmetry 180 μm × 20 mm, 5 μm; Waters, Milford, MA) and subsequently washed with 0.1% (v/v) formic acid at a rate of 15 μl/min for 10 min. The eluted samples were then separated on a C18 analytical column (75 μm × 200 mm, 1.7 μm; Waters, Milford, MA) at 225 nl/min using 0.1% formic acid as solvent A and 0.1% formic acid in acetonitrile as solvent B in a stepwise gradient: 0.1–8% B (0–5 min), 8–25% B (5–185 min), 25–45% B (185–201 min), 45–90% B (201–205 min), 90% B (205–213 min), and 90–0.1% B (213–215 min). The eluting peptides were sprayed into the mass spectrometer with the capillary and cone voltages set to 2.3 kV and 45 V, respectively. The five most abundant signals from a survey scan (400–1300 m/z range, 1s scan time) were selected by charge state, and collision energy was applied appropriately for sequential MS/MS fragmentation scanning (50–1800 m/z range, 1s scan time).

### Data processing and protein identification

For data processing and protein identification the Automated Proteomics Pipeline (APP) was used to analyse the MS data (Malm et al., 2014). APP automates the processing of proteomic tasks such as peptide identification, validation and quantitation from LC-MS/MS data and allows easy integration of multiple separate proteomic tools. The raw MS data file was first processed using the Mascot Distiller software (version 2.4.3.2, Matrix Science, London, UK). The resulting mgf files were subsequently converted into the mzXML file format using msconvert (Kessner et al., 2008). The data were searched against the *Bgh* genome using several search engines in parallel and the following settings: trypsin specific digestion with two missed cleavages allowed; peptide tolerance of 200 ppm; fragment tolerance of 0.5 Da; methylthio on Cys and iTRAQ 4-plex for peptide N-t and Lys used as fixed modifications; oxidized Met and Tyr for iTRAQ 4-plex analysis in variable mode. PeptideProphet was used to validate the results from the searches (Keller et al., 2002).

### Light microscopy

*n*-Hexacosanal/Formvar® resin coated glass slides were inoculated with *Bgh* conidia and moist sponges were placed underneath to maintain humidity. Samples were incubated in the dark and taken out for observation at specific time points (0 h, 2 h, 4 h and 6 h). Fungal structures were then labelled with wheat germ agglutinin (WGA), which selectively binds to *N*-acetylglucosaminyl residues in the fungal cell wall, coupled with Alexa Fluor^TM^-555 (Life Technologies, Carlsbad, CA, USA) following the manufacturer’s protocol. For Calcofluor White labelling, slides were incubated with 300 mM sodium hydroxide (NaOH) for 1 h prior to labelling with 0.01% Calcofluor White for 10 min. The labelled *Bgh* cells were observed using a Carl Zeiss fluorescence microscope (Axio Imager M2; Carl Zeiss; Oberkochen, Germany). Signals from the conjugated WGA were observed with the dsRED filter set at 545/25 nm excitation and 605/70 nm emission wavelengths. Signals from Calcofluor White were observed with the DAPI filter set at 365 nm excitation and 445/50 nm emission wavelengths.

## Results

### Transcriptomic and proteomic analyses

**Fig. 1:**
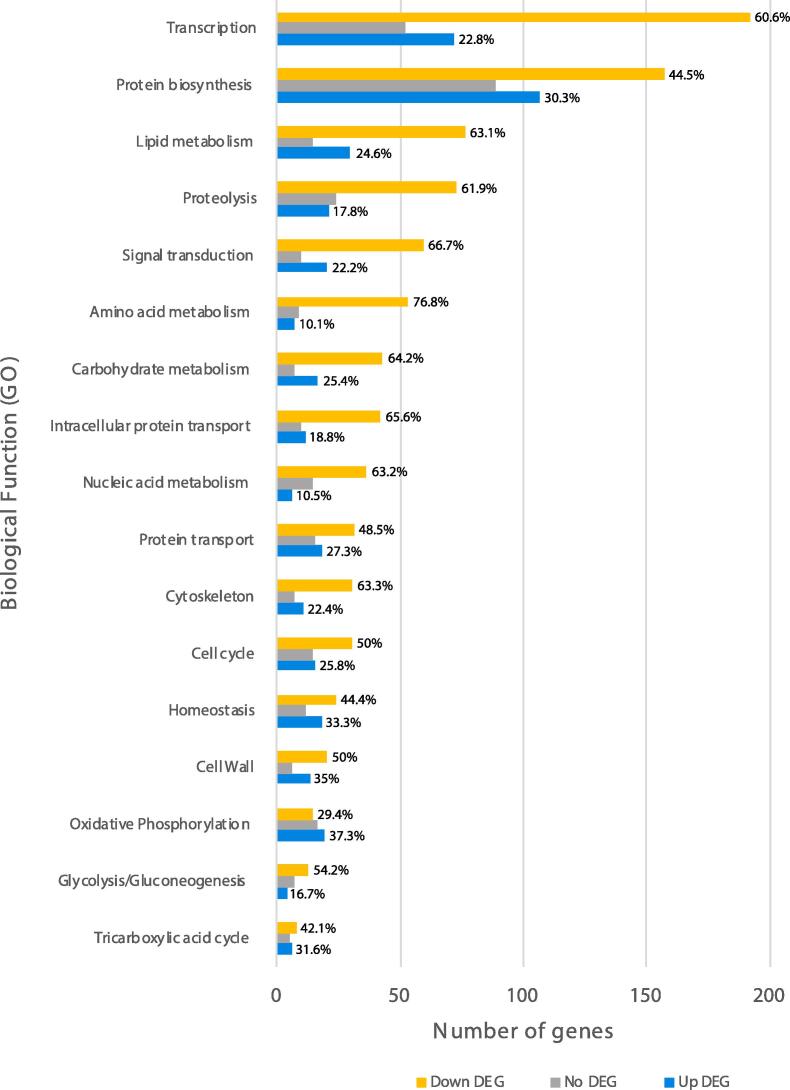
Functional classification of differentially expressed genes (DEG) in *Blumeria graminis* f. sp. *hordei* conidia and during the appressorial germ tube stage. Genes were categorised based on Gene Ontology (GO). Number of genes up-regulated during the appressorial germ tube stage are in blue while the orange bars are the genes that are down regulated. Genes that displayed relatively stable expression during both stages are in grey. The percentage of up DEG and down DEG in each category relative to the total proportion are included in the graph.

**Fig. 2:**
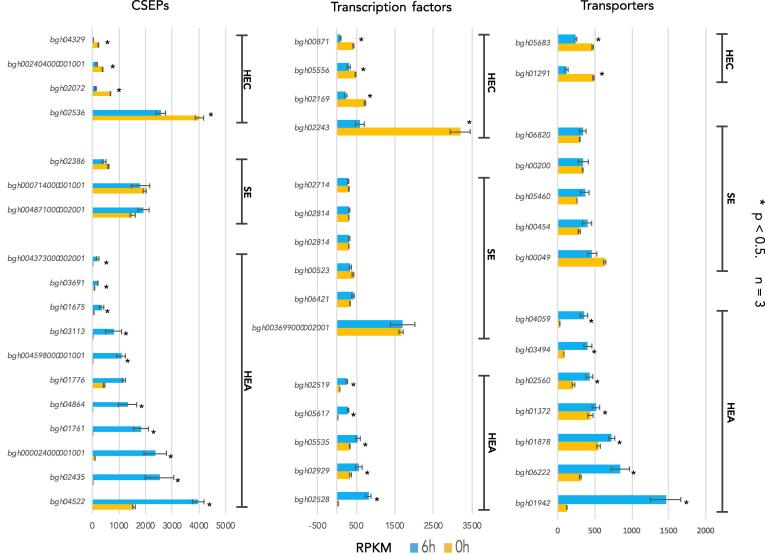
Highest expressed candidate secreted effector proteins (CSEP), transporters, and transcription factor transcripts in the *Blumeria graminis* f. sp. *hordei* conidia (0 h) and agt stage (6 h). HEC, high expression in conidia. SE, stable expression in the conidia and agt. HEA, high expression in the agt. Data are shown as means of three replications ± SD. A paired t-test was done to determine statistical significance.

The expression profile of *Bgh* during the pre-penetrative phase of pathogenesis was investigated by analysing the transcriptomes of the ungerminated *Bgh* conidia and agt stage using Illumina RNA sequencing (Supplementary Table S2). Genes were sorted into functional categories based on Gene Ontology (GO) as shown in Fig. 1. A paired t-test identified the genes differentially expressed between the *Bgh* conidia and agt stage. The categories which had the largest number of differentially expressed genes during the agt stage were transcription, protein biosynthesis, and lipid metabolism. Overall there was a switch towards energy and protein production during the agt stage, with a large up-regulation of genes involved in cellular respiration and protein biosynthesis. Key regulators in glycolysis such as phosphofructokinase (*bgh00033*) and pyruvate kinase (*bgh00037*) were up-regulated while regulatory enzymes in gluconeogenesis, such as glucose 6-phosphatase (*bgh05280*), fructose 1,6-bisphosphatase (*bgh02549*), phosphoenolpyruvate carboxykinase (*bgh06596*), and pyruvate carboxylase (*bgh00049*) were down-regulated (Supplementary Fig. S1). Similarly, many of the genes up-regulated in the lipid metabolism group were involved in lipid and fatty acid catabolism while the vast majority of down-regulated genes were associated with lipid, fatty acid, and long chain fatty acid biosynthesis. Genes involved in oxidative phosphorylation, such as ATP synthases and ATPases, were also highly up-regulated (Fig. 2).

The most up-regulated genes were those involved in transcription and protein biosynthesis. The differential expression between the two developmental stages indicates that there is a shift towards pathogenic specific transcription and translation factors and complexes. A similar gene to the *Neurospora crassa* bZIP transcription factor *HacA* (*bgh02519*) was up-regulated during the agt stage (Fig. 2). *NcHacA* is an important transcription factor to the unfolded protein response (Montenegro-Montero et al., 2015) is activated when the folding capacity of the endoplasmic reticulum is exceeded, such as during germination when enzymes are secreted. In addition, a gene similar to the *Saccharomyces cerevisiae Msn2* C_2_H_2_ transcription factor *Msn2* (*bgh05556*) displayed relatively high expression during both developmental phases. *Msn2* is a general stress response transcription factor of the high osmolarity glycerol (HOG) pathway. Orthologues of *Msn2* have been found to be important for hyphal growth, conidiation, and virulence in several pathogenic fungi (Zhang et al., 2014, Luo et al., 2015, Tian et al., 2017).

**Fig. 3:**
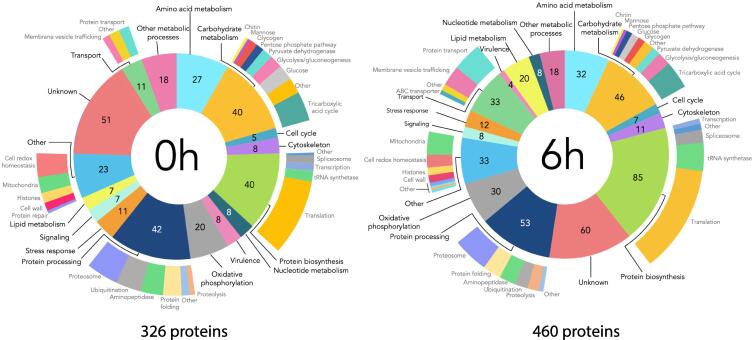
Pie chart representing the functional classifications of the identified proteins from *Blumeria graminis* f. sp. *hordei* conidia and appressorial germ tube stage. The diagram shows the number of proteins identified in various functional categories based on their GO annotations. The outer layer of the pie chart provides additional information to some of the broader categories.

To complement the transcriptome analyses, the proteomic profile of *Bgh* was analysed during the conidial and agt stages. The identified proteins were sorted into broad categories based on their GO annotations. A total of 542 proteins were identified, with 326 proteins detected in the ungerminated conidia (0 h) and 460 detected at the agt stage of *Bgh* (6 h) (Fig. 3). Two hundred and fourty four of these proteins were common to both stages while 82 and 216 were unique to the conidia and agt stage, respectively. The majority of proteins identified were classified as metabolic proteins, representing approximately 60% of total proteins during both the conidial and agt stages. Many of the metabolic proteins identified were involved in amino acid, carbohydrate, lipid, nucleotide, and protein metabolism. Similar to the transcriptomic analysis, a transition towards energy and protein production during the agt stage was observed in the proteomic profile of *Bgh*. The largest number of proteins identified, aside from those of unknown function, are represented by protein metabolism and modification as well as carbohydrate metabolism. The amount of proteins involved in oxidative phosphorylation increased by 1.5-fold during the agt stage while those involved in protein biosynthesis increased by 2-fold. The greatest shift in proteins identified were those involved with lipid metabolism and protein transport, which increased by 3-fold. In regard to transport proteins, there was a high increase in proteins involved in membrane vesicle trafficking and protein transport.

Several candidate secreted effector proteins (CSEPs) are differentially expressed during the conidial and agt stages (Fig. 2). The majority of the highest expressed CSEPs were the same as the highest expressed transcripts measured in *Bgh* cultivated *in planta* on *Arabidopsis* at 6 h (Fig. 2) (Hacquard et al., 2013). In addition, several CSEPs were also found to be highly expressed in the conidia (*bgh02536, bgh02072, bghG002404000001001* and *bgh04329*). The CSEPs detected in the *Bgh* proteome did not correlate well with the transcriptomic data. While eight CSEP proteins were detected, their transcripts were detected at low levels during both developmental stages, apart from CSEP0041 (*bgh02072*) (Supplementary Table S2).

Several transporters involved in multidrug resistance were also detected in the transcriptome and proteome. During the conidial stage, the transporters with the highest transcript expression were two putative major facilitator superfamily (MFS) multidrug transporters (*bgh01291* and *bgh05683*) (Fig. 2). Additionally, a few stage specific ATP-binding cassette (ABC) transport proteins were detected during the agt stage (*bgh01226, bgh00933*, and *bgh01627*). ABC and MFS proteins are among the most prominent contributors to multidrug resistance and virulence, playing important roles in antibiotic-mediated interactions between bacteria and fungi in plant-associated environments (Schoonbeek et al., 2002, Costa et al., 2014).

### Compositional analysis of the *Bgh* cell wall

**Fig. 4:**
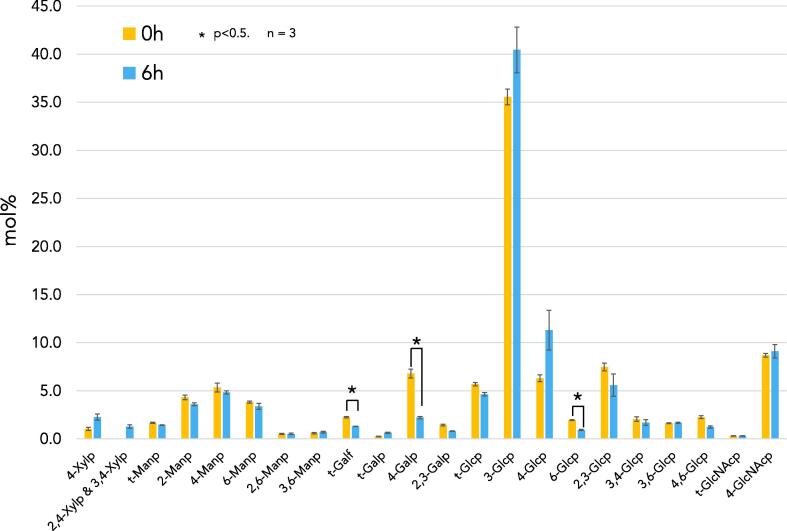
*Blumeria graminis* f. sp. *hordei* glycosidic linkage analysis (mol%) of the carbohydrate fraction of the cell wall from ungerminated conidia (0 h) and the appressorial germ tube stage (6 h). The different glycosidic linkages and pyranose or furanose forms of each monosaccharide were deduced from EI-MS spectra. Gal, galactose; Glc, glucose; GlcNAc, *N*-acetylglucosamine; Man, mannose; Xyl, xylose; ’p’ and ’f’ at the end of a monosaccharide abbreviation indicate that the residue occurs in the pyranose or furanose form, respectively; ‘t-’ indicates a ‘terminal’ monosaccharide that occurs at the non-reducing end of a glycan. Error bars indicate standard deviations calculated from three biological replicates. A paired t-test was done to determine statistical significance.

Analysis of the cell wall polysaccharides of ungerminated *Bgh* conidia (Pham et al., 2019) and agt stage revealed that a majority of the fungal cell wall polysaccharides were composed of glucosyl, *N*-acetylglucosaminyl, mannosyl, galactosyl, and trace amounts of xylosyl residues (Fig. 4). The total amount of glucosyl, mannosyl, and *N*-acetylglucosaminyl residues remained relatively stable between the two developmental stages. However, while the proportions of the mannosyl and *N*-acetylglucosaminyl linkage types were the same, there were some changes in the proportion of glucosyl linkage types. Compared to the conidial stage, the 1,3- and 1,4-linked glucosyl residues increased by 12.8% and 57% respectively during the agt stage, while the 1,6-linked, 1,2,3 and 1,3,4-linked residues decreased by 70.4%, 29%, and 18.4%, respectively.

The proportion of galactosyl residues in the cell wall exhibited the most dramatic decrease during the agt stage, with a total decrease of 73.5% compared to the conidial stage. The terminal galactofuranosyl residues were reduced by 53% while 1,4-linked galactopyranosyl units decreased by 101.7%. The overall proportion of xylosyl residues increased by 3-fold. The 1,4-linked xylosyl residues increased by 2-fold during the agt stage and the presence of 2,4- and 3,4-linked xylosyl residues indicative of the presence of branching points was detected specifically during the agt stage.

### *Bgh* cell wall labelling

**Fig. 5:**
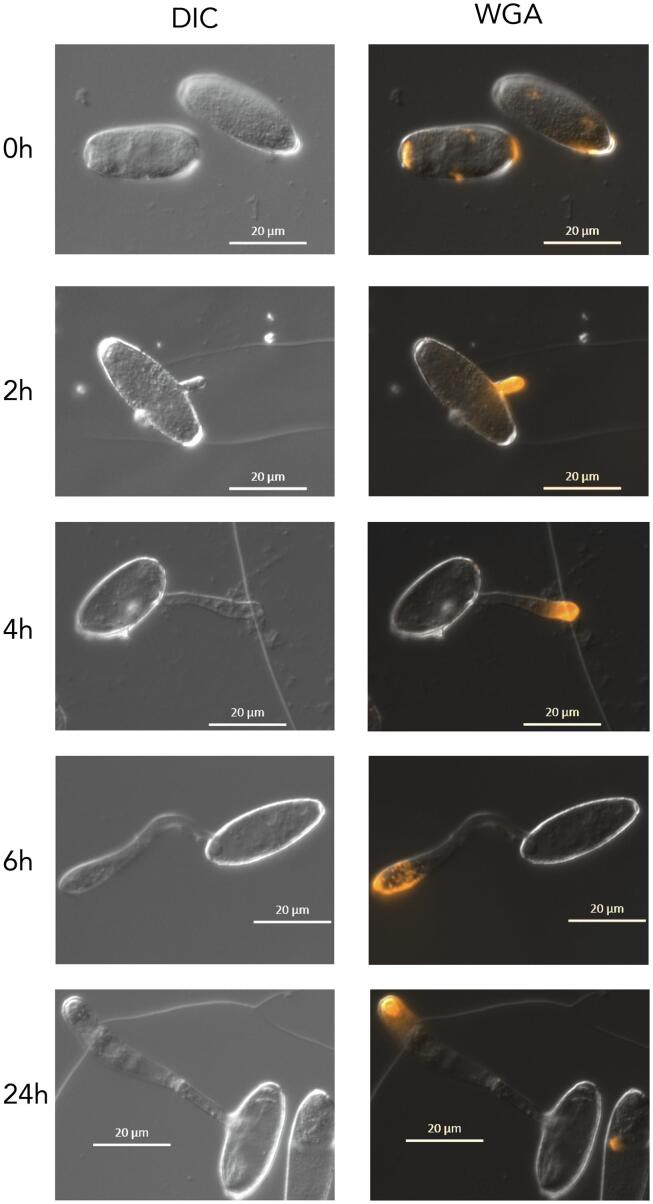
Light microscopy of *Blumeria graminis* f. sp. *hordei* development on glass slides coated with *n*-hexacosanal/Formvar® resin labelled with wheat germ agglutinin (WGA) conjugated to Alexa Fluor 555 (WGA-AF555). 0 h: Conidium immediately after inoculation, 2 h: emergence of primary germ tube (pgt), 4 h: development of secondary germ tube (sgt), 6 h: differentiation of sgt to appressorial germ tube (agt), 24 h: development of a penetration peg under the appressorium. DIC, differential interference contrast; dsRED filter to observe WGA-AF555 labels.

Developing *Bgh* cells were labelled with the chitin-binding probes, WGA and Calcofluor White. Calcofluor White is a fluorescent stain known to strongly bind to chitin while WGA is a lectin that binds to the *N*-acetylglucosaminyl residues that compose the chitin in fungal cell walls. WGA was applied directly to the germinating *Bgh* cells and bound to newly developing structures (Fig. 5). On freshly inoculated slides (0 h) WGA labelled the buds scars at the ends of the conidia, though over time it can be observed that the labelling of the scars dissipates (2–6 h). *Bgh* conidia develop on a structure called conidiophores, a chain of linear conidia connected to each other. Bud scars form between the conidia during conidiation, an asexual reproduction process in filamentous fungi. As the conidia develop, the connections constrict and eventually weaken to the point that they can be broken by wind stress, releasing the conidia from the conidiophore and exposing the scars.

**Fig. 6:**
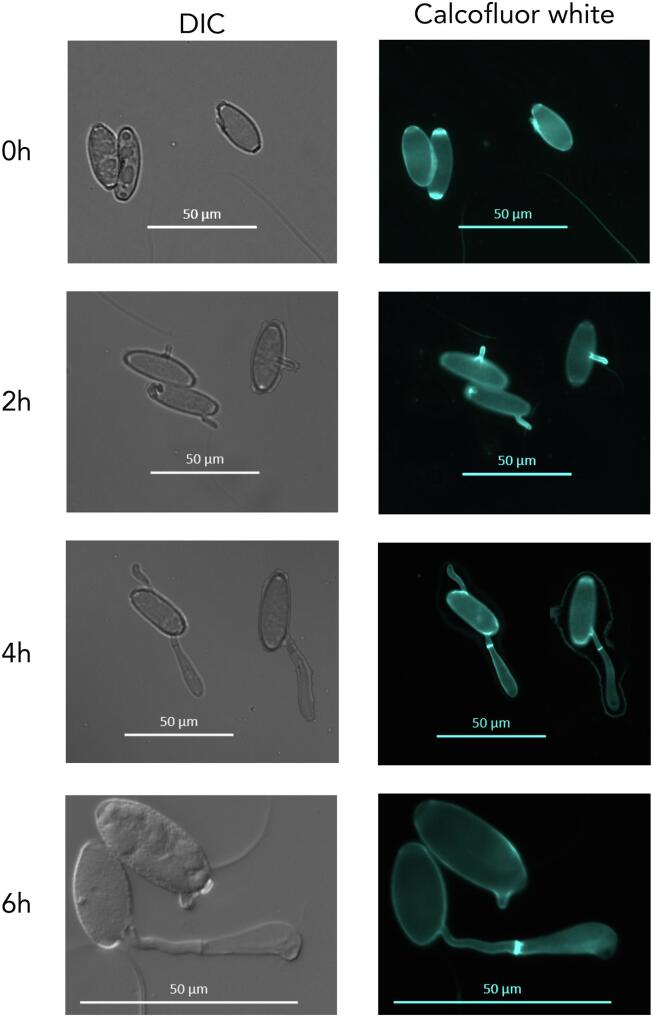
Light microscopy of *Blumeria graminis* f. sp. *hordei* development on glass slides coated with *n*-hexacosanal/Formvar® resin labelled with Calcofluor White. 0 h: Conidium immediately after inoculation, 2 h: emergence of primary germ tube (pgt), 4 h: development of secondary germ tube (sgt), 6 h: differentiation of sgt to appressorial germ tube (agt). At 4 h and 6 h a septum is present between the conidium and sgt/agt. DIC, differential interference contrast; DAPI filter to observe Calcofluor White.

At 2 h WGA binds to newly emerged primary germ tubes. However, after 2 h it appears that WGA does not bind to the primary germ tubes anymore. In fact, at 4 h and 6 h the WGA only labels the tips of the developing secondary germ tubes and agt, respectively. After 24 h WGA only labels the *Bgh* cells that developed a penetration peg under the appressorium. From these observations, it can be concluded that WGA is only able to label newly emerging structures and that the cells mask any exposed chitin over time as these structures are no longer labelled. To label the internal structures, the developing *Bgh* cells were permeabilised with NaOH so that Calcofluor White could penetrate through the cell wall. This allowed Calcofluor White to label the entire *Bgh* structures, from the conidia to the developing germ tubes (Fig. 6). Similar to WGA, the bud scars are seen at 0 h and then recede over time. The internal labelling reveals the septum that forms in the secondary germ tubes (4 h) and assists in the development of the agt (6 h).

### *Bgh* cell wall metabolism

**Fig. 7:**
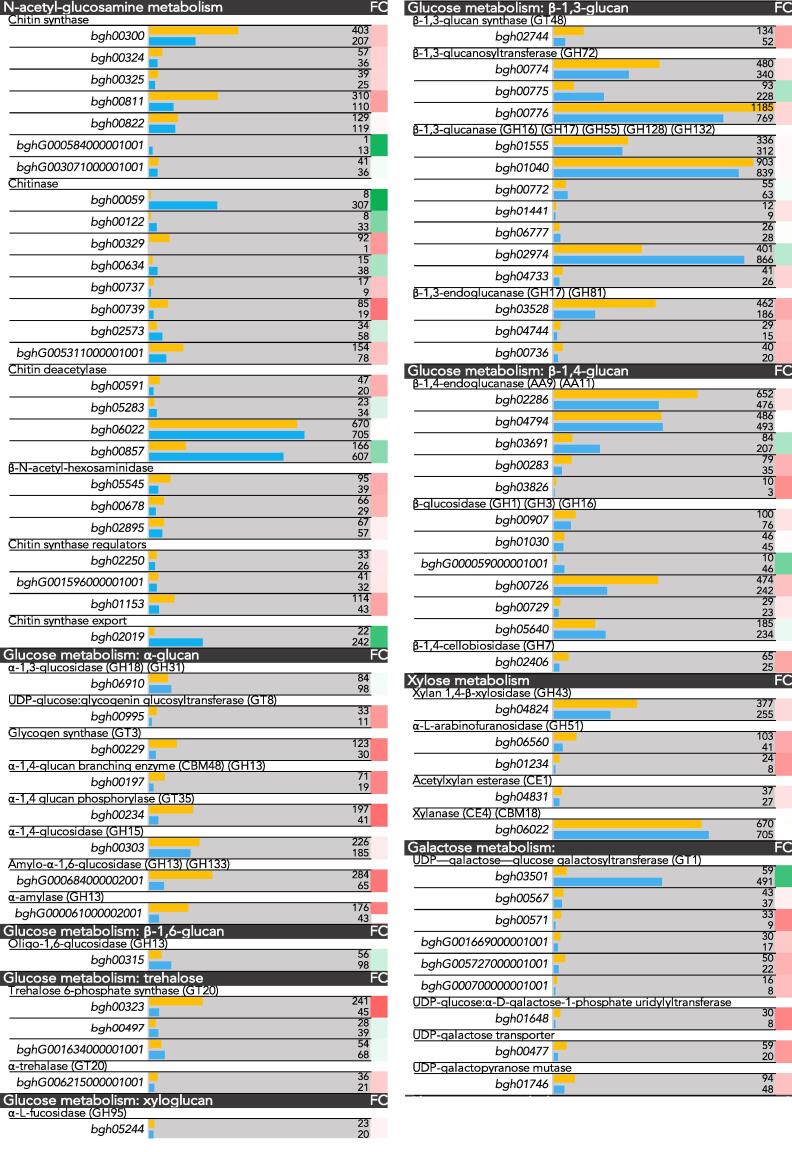

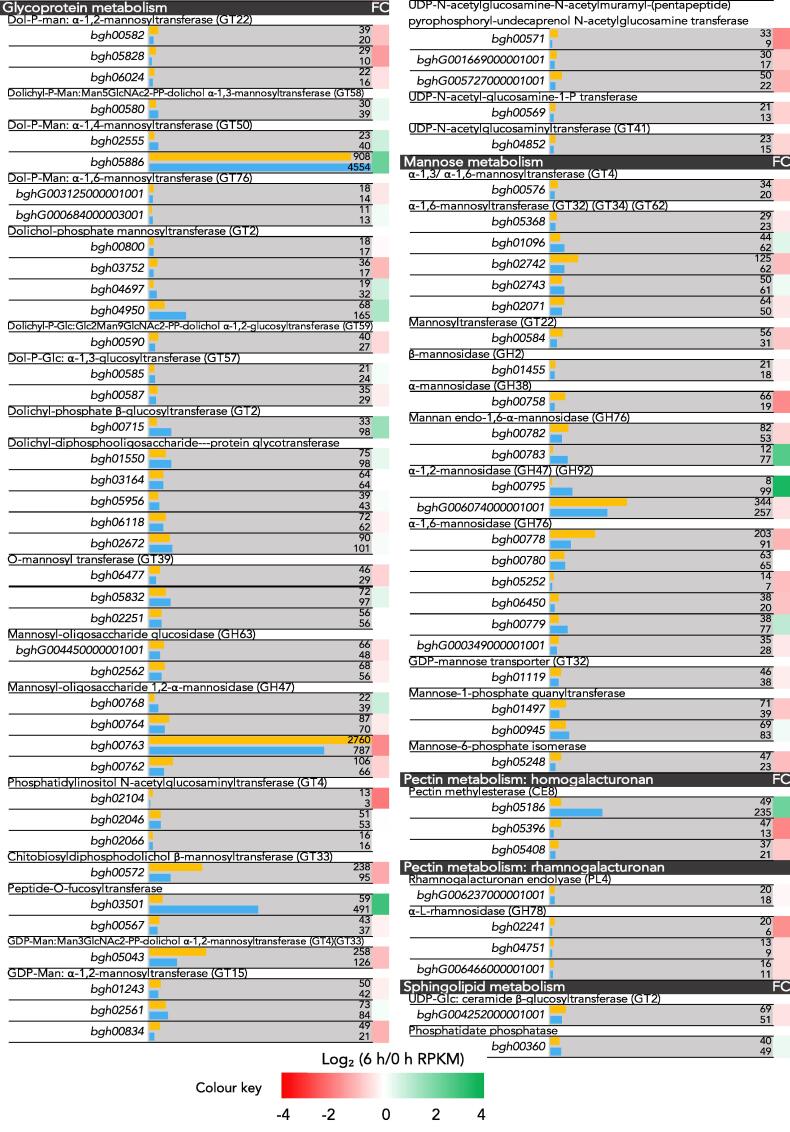
RNA-Seq transcript levels of genes involved in cell wall metabolism in *Blumeria graminis* f. sp. *hordei* conidia (0 h, orange) and during the appressorial germ tube stage (6 h, blue). Genes are organised based on which part of the cell wall metabolism they are involved in. Transcript levels are displayed adjacent to the accession numbers of the genes as RPKM values. Scale of the bar is 0–1000 RPKM, with a few outliers exceeding 1000. FC, log_2_ fold change between the conidia (0 h) and appressorial germ tube stage (6 h).

A table displaying the transcriptomic profile of genes involved in cell wall metabolism was constructed (Fig. 7). The classifications of the genes were based on CAZy and KEGG annotations. Aside from a few exceptions, there was a general down-regulation of genes involved in cell wall metabolism.

Only a few genes, such as chitinase (*bgh00059*), chitin deacetylase (*bgh00857*), β-1,3-glucanase (*bgh02974*), pectin methytransferase (*bgh05186*), and galactosyltransferase (*bgh03501*) displayed increased expression profiles during the agt stage. A UDP-glucose/galactose transporter was also up-regulated during the agt stage (*bgh04059*) (Fig. 2). These genes may play an important role in cell wall development during germination. Several genes maintained high expression during both the conidial and agt stages, such as chitin deacetylase (*bgh06022*), Dol-P-Man: α-1,4-mannosyltransferase (*bgh00763*), β-1,3-glucanase (*bgh01040*), and β-1,3-glucanosyltransferase (*bgh00776*), indicating possible key roles in cell wall maintenance and development during early developmental stages.

A few agt-specific proteins involved in cell wall processes were detected as well, such as the cell wall biogenesis protein phosphatase SSD1. SSD1 is an important regulator of fungal cell wall biogenesis and composition, and is also important in mediating resistance to osmotin in a cell wall-dependent manner in *S. cerevisiae, Colletotrichum lagenarium*, and *Magnaporthe grisea* (Ibeas et al., 2001, Tanaka et al., 2007). Osmotin is a plant defence antifungal protein belonging to the pathogenesis-related (PR) 5 family. Additionally, the chitinase with high expression in the transcriptome (*bgh00059*) was also detected in the proteome. Chitinases have been implicated in assisting cell wall growth by plasticising the cell wall (Tzelepis et al., 2012).

**Fig. 8:**
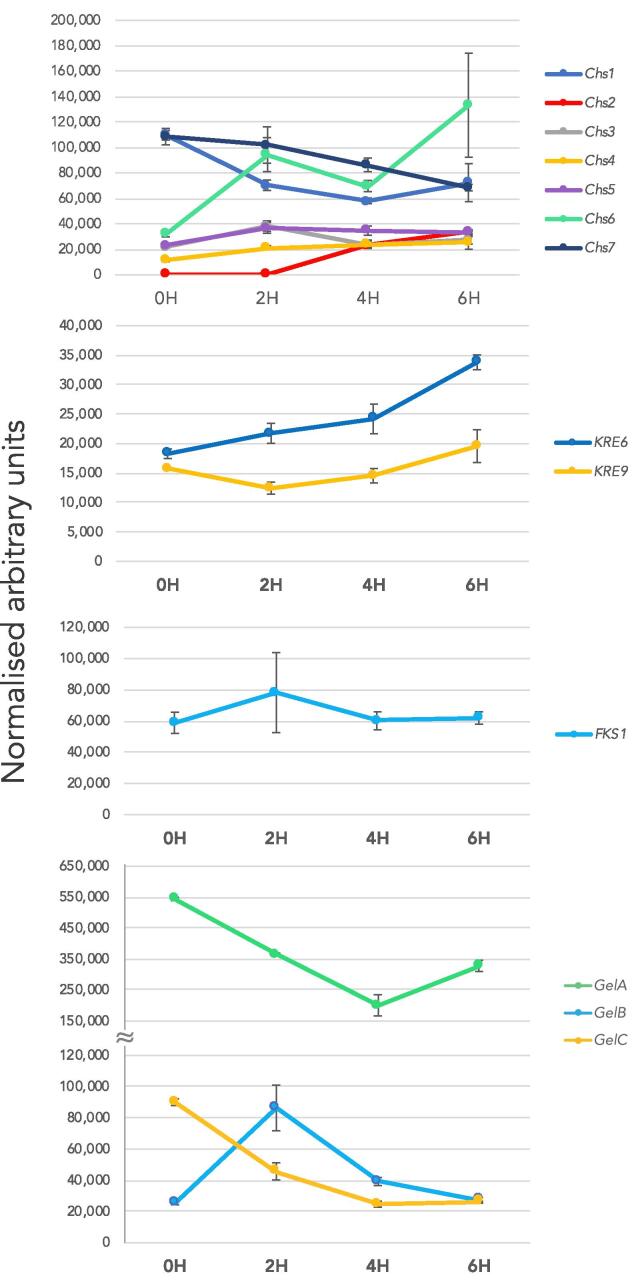
Transcript profile (qPCR) of cell wall related genes in *Blumeria graminis* f. sp. *hordei* cells cultivated *in vitro* during early germination (0–6 h). Data are shown as means of three replications ± SD.

The expression profile of enzymes involved in chitin (*Chs1-7*) and β-1,3-glucan (*Fks1, GELA/B/C, KRE6* and *KRE9*) biosynthesis and remodelling was analysed by qPCR during the pre-penetrative phases of *Bgh* pathogenesis (0 h, 2 h, 4 h and 6 h) (Fig. 8). The chitin synthase genes displayed variable profiles over the course of 6 h. While the expression of some genes remained stable (*Chs3, 4* and *5*), the expression of others decreased (*Chs1* and *7*). The only chitin synthases that were up-regulated were *Chs2* and *Chs6*. *Chs2* was not expressed at 0 h and 2 h, and only begun to display expression at 4 h with an increased level at 6 h. This coincided with the formation of the septum in the secondary germ tube at 4 h and could possibly be associated with septum formation and development (Fig. 6). *Chs6* expression increased over the 6 h time course, which suggests an involvement of this gene in germination and secondary/appressorial germ tube development.

There was a general down regulation of the β-1,3-glucanosyltransferases (*GELA/B/C*) over time, though *GELA* was still the highest expressing gene over the course of 6 h. *GELB* expression peaked at 2 h and then decreased. This may indicate that *GELB* is involved in primary germ tube development. The expression of the β-1,3-glucan synthase (*Fks1*) and the putative β-1,6-glucan synthase (*KRE9*) remained relatively stable (Fig. 8) while the *KRE6* exhibited a general trend of up-regulation, indicating a possible involvement in early development.

The qPCR results were used to validate the RNA-Seq dataset. A good correlation was observed between the log_2_ of the fold change ratios (6 h/0 h) of the RNA-Seq and qPCR expression profiles ($y=0.6659x-0.859\text{;}R^{2}=0.8973$), indicating the reliability of the RNA-Seq dataset (Supplementary Fig. S2).

## Discussion

Characterising the complex physiological and molecular changes that occur during pathogenesis is an important step towards disease control. As an obligate biotroph the range of molecular techniques applicable to *Bgh* is limited, as it cannot be cultured *in vitro*. In this study, we used a method adapted from Hansjakob et al. (2010) to cultivate *Bgh in vitro* up to the pre-penetrative stages of pathogenesis to examine the physiological and molecular shifts that occur during germination by analysing the transcriptome, proteome, and cell wall composition.

Transcriptomic analysis of the metabolic processes in *Bgh* reveals that there is a shift towards energy and protein production during the agt stage. After 6 h many genes involved in cellular respiration and protein synthesis were up-regulated. Key regulators in glycolysis and enzymes involved in lipid catabolism were up-regulated during the agt stage. This coincided with the expression profile of *Bgh* primary metabolism previously conducted by Both et al. (2005), where an increase in glycolytic processes and lipid metabolism during pre-penetrative development was observed. The processes of glycolysis and lipid metabolism consist of catabolic activities that generate energy to make use of the glycogen and lipid droplets stored in the *Bgh* conidia (Both et al., 2005). Several enzymes involved in oxidative phosphorylation were also up-regulated. The majority of the highest expressing transporters were various ATP synthases and ATPases that were also highly up-regulated at 6 h *in planta* on *Arabidopsis* (Hacquard et al., 2013).

Genes involved in transcription and protein synthesis were by far the most up-regulated during the agt stage. Several of the transcription factors up-regulated seemed to be important for virulence. The *Msn2* transcription factor (*bgh05556*) was highly expressed during both the conidial and agt stages of development. Orthologues of *Msn2* have been found for be important to hyphal growth and virulence in other pathogenic fungi (Zhang et al., 2014, Luo et al., 2015, Tian et al., 2017). The *N. crassa hac-1* has an important role in aiding the secretion of enzymes during ER stress conditions (Montenegro-Montero et al., 2015). The putative *Bgh* orthologue *HacA* (*bgh02519*) was up-regulated during the agt stage and likely assists in the secretion of plant cell wall degrading enzymes that facilitate the penetration process.

The proteome from the agt stage supports the results from the transcriptomic analysis. During the agt stage, there was a significant increase in proteins involved in lipid metabolism and protein biosynthesis, processing, and transport. There was a greater increase in proteins involved in lipid metabolism than glycolysis. This was likely more energy efficient as, compared to carbohydrates, the breakdown of fatty acids yields more ATP during complete oxidation by the Lynen helix and tricarboxylic acid cycle. The sheer volume of proteins involved in protein biosynthesis points to a rapid need to generate more proteins during pathogenesis. This is accompanied by a significant increase in proteins associated with membrane vesicle trafficking and protein transport.

As the agt stage precedes penetration it is likely that a great supply of energy is necessary to generate the turgor pressure in the appressoria to penetrate the plant cell wall and to also fuel the synthesis of new proteins for virulence factors and the development of the haustoria.

*Bgh* is constantly growing and developing new structures throughout pathogenesis. As the fungus grows there is a constant demand for more protein production. Previous proteomic analyses of the *Bgh* conidia, haustoria and sporulating hyphae have shown that proteins associated with protein biosynthesis, modification, and metabolism are present in high levels at all developmental stages (Bindschedler et al., 2009, Godfrey et al., 2009).

A few studies have previously analysed the *Bgh* conidia (Bindschedler et al., 2009, Noir et al., 2009), with some able to analyse the *in planta* proteome of *Bgh* haustoria and sporulating hyphae thanks to the sequencing of the *Bgh* genome (Bindschedler et al., 2009, Godfrey et al., 2009). The majority of the proteins identified in the conidia were classified as metabolic proteins (62%). Metabolic proteins are generally well represented in proteomic studies as they are highly abundant and soluble. *Bgh* is not the only phytopathogenic fungus to exhibit this profile in the conidia as spores from the fungal plant pathogen *Uromyces appendiculatus* also mainly consisted of metabolic proteins (66%) (Cooper et al., 2006). It has been suggested that asexual fungal spores preform metabolic proteins to be prepared to rapidly produce proteins upon contact with the host surface without expending the cell limited energy resources on *de novo* assembly (Cooper et al., 2006, Loginov and Šebela, 2016).

Pathogens are able to suppress the host innate immune mechanisms and manipulate host cellular functions to redirect nutrients by secreting effector molecules (Win et al., 2012). In *Bgh*, this is achieved by CSEPs. Over 500 CSEPs have been identified in *Bgh* (Spanu et al., 2010), though only a few have been shown to play a role in virulence (Aguilar et al., 2016, Ahmed et al., 2016). Analysis of the CSEP transcripts reveals that several CSEPs are highly expressed in the conidia and agt stages. Hacquard et al. (2013) examined the transcript profile of CSEPs from *Bgh* infected *Arabidopsis* from 6 h onward. The majority of the highest expressed CSEPs in their study were the same as those detected on the *in vitro* surface, providing confidence that the *in vitro* surface is stimulating similar transcript profiles as *in planta*. In addition, several CSEPs were found to be highly expressed in the *Bgh* conidia. However, apart from CSEP0041 (*bgh02072*), none of the highly expressed CSEPs were detected in the proteomic analysis. Seven CSEPs were detected in the conidia and expressed at low levels during both developmental stages (*bgh05118, bgh02337, bgh04522, bgh03855, bgh05117, bghG003669000001001*, and *bgh00804*) (aside from CSEP0041). These conidia-specific CSEPs are likely involved in the early stages of pathogenesis and are possibly secreted in the extracellular matrix that is released when *Bgh* conidia land on a plant surface. Of these 7 CSEPs, four were also detected during the agt stage (*bgh05118, bgh02072, bgh02337*, and *bgh04522*) and possibly have roles in interfering with important plant processes during host penetration or haustorium development.

Glycosidic linkage analysis of the *Bgh* cell wall fraction allowed the identification of its constituent polysaccharides (Fig. 4). Previous analysis of the *Bgh* conidial cell wall polysaccharides revealed that they were primarily composed of glucosyl residues, had a greater proportion of galactopyranosyl residues compared to other species, and also contained 1,4-linked xylosyl residues (Pham et al., 2019). After 6 h there was a shift in the amount of glucosyl linkages, with an increase in 1,3- and 1,4-linked glucosyl residues and a decrease in 1,6-linked residues. While the galactosyl portion of the cell wall experienced a large decrease in content (54%), its function is unknown as the biosynthesis of a galactose-containing polymer as well as its biological role and importance for fungal viability are currently undefined. The xylosyl proportion of the cell wall increased by 3-fold, with the presence of residues indicative of branching points and the proportion of 1,4-linked xylosyl residues doubling. The presence of branched 1,4 xylosyl residues is novel and has not yet been reported in fungi. It was previously suggested that xylosyl residues in the *Bgh* cell wall may form xylomannan, an antifreeze molecule (Pham et al., 2019), as xylomannan has been detected in the cell walls of other fungal species (Walters et al., 2011, Kawahara et al., 2016).

The agt stage consists of a secondary germ tube emerging from the side of the *Bgh* conidial cell that has differentiated into an appressorium (Fig. 6, 6 h). The germ tube itself would contribute less than 5% of the total cell wall mass, with minimal changes expected to occur compared to the composition of the cell wall of the ungerminated conidium. Therefore, it may be possible to extrapolate the composition of the germ tube from the difference between the conidial and agt stages. The cell wall of the germ tube would likely have a chitin and β-1,3-glucan foundation, like the majority of fungal cell walls. As there was an increase in 1,3- and 1,4-linked glucosyl residues as well as branch-type and 1,4-xylosyl residues, it can be inferred that the majority of the new polysaccharides synthesised would be in the agt.

In terms of cell wall metabolism, some of the highest expressed genes encode chitinase (*bgh00059*) and chitin deacetylases (Fig. 7). The expression of these genes could be an important element during pre-penetrative development. Not only was chitinase highly expressed but the protein was also detected during the agt stage. Chitinases have been shown to play an important role during polar growth and have been suggested to have a plasticising function in *N. crassa* during hyphal development (Tzelepis et al., 2012).

Chitin deacetylases are important for virulence as they catalyse post-synthetic modifications of chitin by deacetylating it to form chitosan. Chitosan is a weak inducer of the host defence system and assists fungi in evading the host response (Fujikawa et al., 2012, Hadwiger, 2013). Labelling the chitin of the cell walls revealed that while exposed bud scars and newly emerging structures are labelled, over time WGA ceased binding to these structures as they were most likely swiftly deacetylated to chitosan to avoid triggering plant immunity (Fig. 6).

Chitin deacetylases have recently been associated with inducing appressorium differentiation in *Magnaporthe oryzae* (Kuroki et al., 2017). It has been proposed that chitin deacetylases not only function in avoidance of the host defence system, but are also involved in signalling, as exogenous sources of chitosan were able to induce appressorial formation in *M. oryzae* (Geoghegan and Gurr, 2016). In addition, chitin deacetylase deletions in *M. oryzae* resulted in compromised appressoria. The chitin deacetylases expressed during the agt stage could be an important factor for pathogenesis and agt development in *Bgh*.

Analysis of the expression of the *Bgh* chitin synthases by qPCR over 6 h revealed that CHS2 may be involved in septum formation, as its expression coincides with the formation of the septum in the agt, while CHS6 may contribute to germ tube development, as it was the only CHS enzyme to be up-regulated over 6 h (Fig. 6 and 8). Class II chitin synthases, such as *Bgh* CHS2, have been associated with septum formation and lateral cell wall integrity in *Candida albicans* (Roncero, 2002). The *Bgh* CHS6 is orthologous to the *Ustilago maydis* CHS6, which has been found to be important in elongating hyphal tips and co-transporting β-1,3-glucan synthase to sites of growth (Schuster et al., 2016). Both CSH2 and CHS6 may hold important roles in cell wall development during early pathogenesis.

*Bgh* is one of the most destructive foliar pathogens of barley. Despite the economic and agricultural significance of *Bgh*, molecular analysis of the pathogen has fallen significantly behind that of other filamentous pathogenic fungi, with a gap in knowledge surrounding the events during appressorial development. Due to its nature as an obligate biotroph the range of molecular techniques that can be carried out on *Bgh* is limited.

This study demonstrates that it is possible to cultivate *Bgh in vitro* up to the pre-penetrative stage of development to generate fungal material for molecular analysis without the problems associated with *in planta* studies. Transcriptomic profiling of the glycolytic processes, lipid metabolism, transcription factors, transporters, and virulence factors (CSEPs) also corroborate with *in planta Bgh* expression profiles from previous studies (Both et al., 2005, Hacquard et al., 2013), demonstrating that pre-penetrative *in vitro* events can serve as a viable approximation for pre-penetrative *in planta* events.

## Author’s contributions

Conceived and designed the experiments: TP VB AL. Performed the experiments: TP JS NS XX YS VS. Analyzed the data: TP JS NS XX VS VB AL. Contributed reagents/materials/analysis tools: VB. Wrote and edited the paper: TP AL VB.

## Funding

This work was supported by the Australian Research Council Centre of Excellence in Plant Cell Walls (CE110001007).

## Declaration of Competing Interest

The authors declare that they have no known competing financial interests or personal relationships that could have appeared to influence the work reported in this paper.

## Supplementary data

Supplementary Fig. S1 Diagram of the metabolic pathway of glycolysis and the relative expression of the corresponding enzymes during the appressorial germ tube stage of *Blumeria graminis* f. sp. *hordei* development. Glycolysis is the process in which glucose is converted to pyruvate. Key glycolytic enzymes are highlighted by the blue text and arrows. Gluconeogenesis is the opposite of glycolysis and is a process that results in the generation of glucose. As it is the reverse process, glycolysis and gluconeogenesis share many enzymes that have reversible activity. Key enzymes in gluconeogenesis are denoted by the orange arrows and text. Arrows denote the direction of the enzymatic reaction. Double headed arrows mean that the enzymatic reaction is reversible while single direction arrows indicate that the reaction is irreversible. Enzyme up-regulation and down-regulation are designated by the green and red arrows respectively while stable expression is indicated by tildes.

Supplementary Fig. S2 Correlation of the log_2_ of the fold change (6 h/0 h) between RNA-Seq (RPKM) and qPCR (normalised arbitrary units) from *Blumeria graminis* f. sp. *hordei* conidia (0 h) and appressorial germ tube (6 h).

Supplementary Table S1 Primers for qPCR of *Blumeria graminis* f. sp. *hordei* cell wall related genes.

Supplementary Table S2 Summary of RNA-Seq data of *Blumeria graminis* f. sp. *hordei* during the conidial and agt stages of development on an *in vitro* surface. Supplementary table is an excel spread sheet, this figure displays a portion of the spreadsheet.
