# Supplementary figures and images for: Analysis of cell wall synthesis and metabolism during early germination of Blumeria graminis f. sp. hordei conidial cells induced in vitro
Source: Cell Surf. 2019 Aug 14;5:100030. doi: 10.1016/j.tcsw.2019.100030 (PMC7389524; doi:10.1016/j.tcsw.2019.100030)

# Glycolysis and Gluconeogenesis

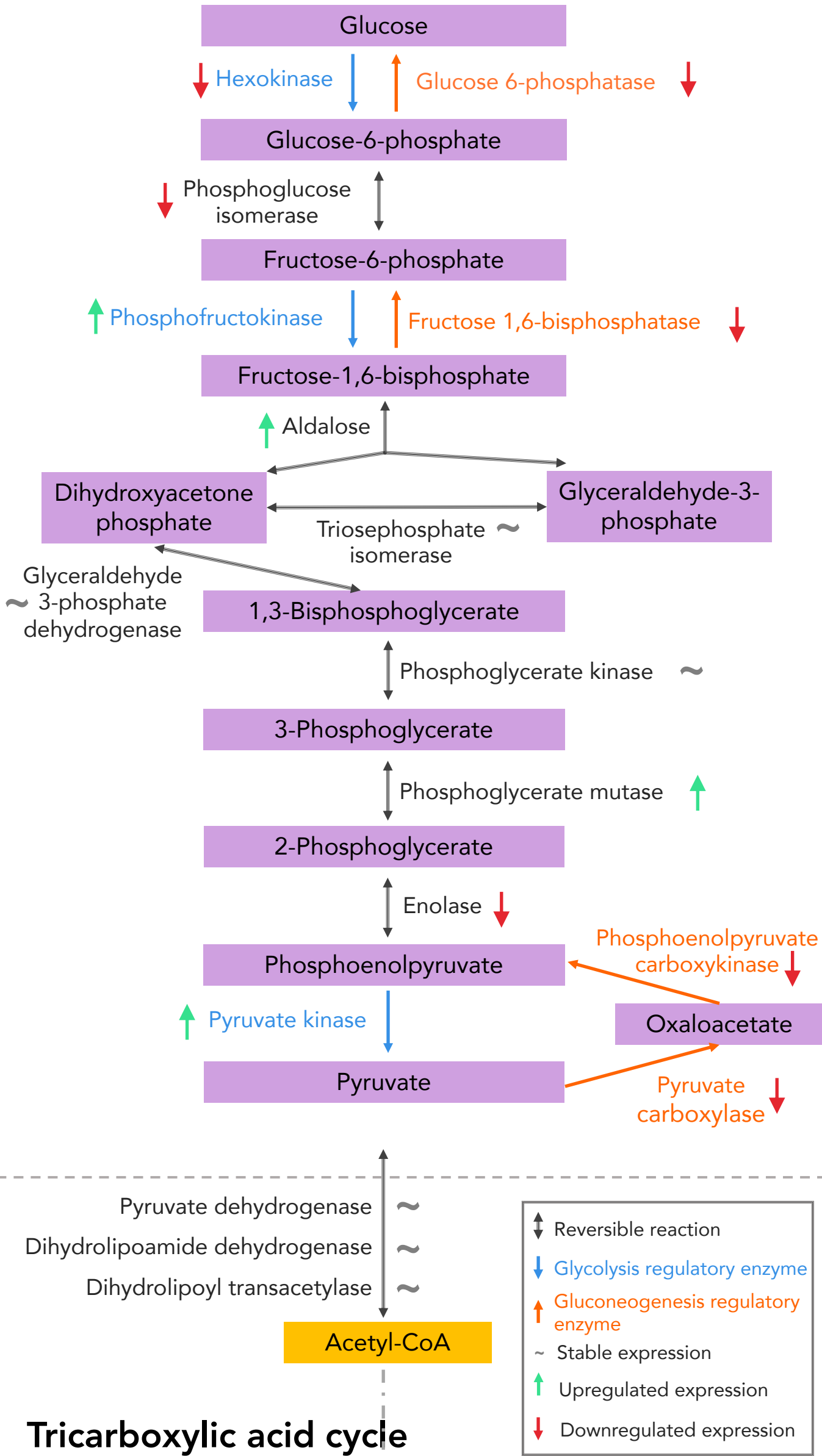

Supplement: Supplementary Fig. S1 — Diagram of the metabolic pathway of glycolysis and the relative expression of the corresponding enzymes during the appressorial germ tube stage of Blumeria graminis f. sp. hordei development. Glycolysis is the process in which glucose is converted to pyruvate. Key glycolytic enzymes are highlighted by the blue text and arrows. Gluconeogenesis is the opposite of glycolysis and is a process that results in the generation of glucose. As it is the reverse process, glycolysis and gluconeogenesis share many enzymes that have reversible activity. Key enzymes in gluconeogenesis are denoted by the orange arrows and text. Arrows denote the direction of the enzymatic reaction. Double headed arrows mean that the enzymatic reaction is reversible while single direction arrows indicate that the reaction is irreversible. Enzyme up-regulation and down-regulation are designated by the green and red arrows respectively while stable expression is indicated by tildes. [file mmc1.pdf]

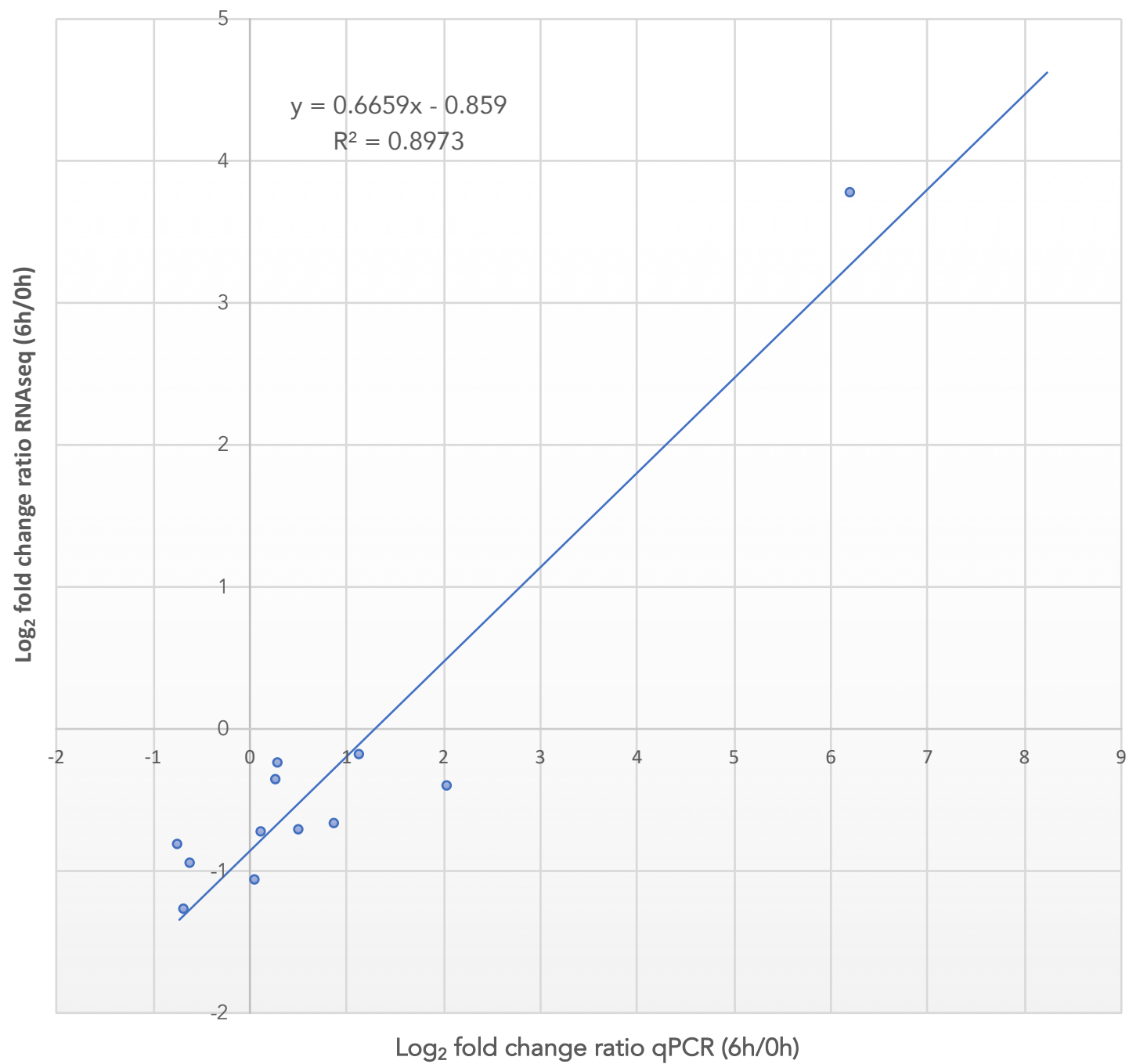

Supplement: Supplementary Fig. S2 — Correlation of the log2 of the fold change (6 h/0 h) between RNA-Seq (RPKM) and qPCR (normalised arbitrary units) from Blumeria graminis f. sp. hordei conidia (0 h) and appressorial germ tube (6 h). [file mmc2.pdf]
